# Changes in Beneficial *C*-glycosylflavones and Policosanol Content in Wheat and Barley Sprouts Subjected to Differential LED Light Conditions

**DOI:** 10.3390/plants9111502

**Published:** 2020-11-06

**Authors:** Muthusamy Muthusamy, Jong Hee Kim, Suk Hee Kim, Joo Yeol Kim, Jeong Wook Heo, HanGyeol Lee, Kwang-Sik Lee, Woo Duck Seo, Soyoung Park, Jin A Kim, Soo In Lee

**Affiliations:** 1 Department of Agricultural Biotechnology, National Institute of Agricultural Sciences (NAS), RDA, Jeonju 54874, Korea; biotech.muthu@gmail.com (M.M.); gllmon@naver.com (J.H.K.); hbhb0@naver.com (S.H.K.); rlawnduf@korea.kr (J.Y.K.); psy0203@korea.kr (S.P.); jakim72@korea.kr (J.A.K.); 2 Division of Horticultural Biotechnology, Hankyung National University, Anseong 17579, Korea; 3 Department of Agricultural Engineering, National Institute of Agricultural Sciences (NAS), RDA, Jeonju 54874, Korea; wooncho@korea.kr; 4 Division of Crop Foundation, National Institute of Crop Science (NICS), RDA, Wanju 55365, Korea; gajae93@gmail.com (H.L.); kslee840118@gmail.com (K.-S.L.); swd2002@korea.kr (W.D.S.)

**Keywords:** saponarin, isoorientin, hexacosanol, octacosanol, fatty acyl-coenzyme A reductase (FAR)

## Abstract

The spectral quality and intensity of light, photoperiodism, and other environmental factors have profound impacts on the metabolic composition of light-dependent higher plants. Hence, we investigate the effects of fluorescent light (96 μmol m^−2^s^−1^) and white (100 μmol m^−2^s^−1^), blue (100 μmol m^−2^s^−1^), and red (93 μmol m^−2^s^−1^) light-emitting diode (LED) light irradiation on the *C*-glycosylflavone and policosanol contents in young seedlings of wheat and barley. Ultra-high-performance liquid chromatography (UHPLC) analyses of *C*-glycosylflavone contents in barley reveal that the saponarin content is significantly enhanced under blue LED light irradiation. Under similar conditions, isoorientin and isoschaftoside contents are improved in wheat seedlings. The contents of these *C*-glycosylflavones differed along with the light quality and growth period. The highest accumulation was observed in sprouts after three days under blue LED light irradiation. GC/MS analyses of policosanol contents showed that 1-hexacosanol (C26:o–OH) in barley and 1-octacosanol (C28:o–OH) in wheat seedlings were reduced under LED light irradiation, compared to seedlings under fluorescent light conditions. Nonetheless, the policosanol contents gradually improved with the extension of growth times and treatments, irrespective of the light quality. Additionally, a positive correlation was observed between the expression pattern of biosynthesis-related genes and the respective metabolite content in barley. This study demonstrates that blue LED light irradiation is useful in maximizing the *C*-glycosylflavone content in barley and wheat sprouts.

## 1. Introduction

Wheat (*Triticum aestivum* L.) is one of the staple food grains for approximately 40% of the global population [1]. It ranks third in terms of global production and its nutritional importance in the human diet has long been investigated [2]. Similarly, barley (*Hordeum vulgare* L.) is the fourth most important cereal crop for both humans and animals worldwide, having the highest dietary fiber content [3,4]. Barley is rich in biologically active molecules/metabolites, which are essential for plants. These metabolites have the potential to exhibit health benefits in the human diet. Barely or its extracts have shown powerful antioxidant effects as dietary supplements for humans. These antioxidant effects are mainly attributable to the presence of saponarin, lutonarin, and hexacosanol molecules [3]. Barley grass also possesses numerous other phytonutrients, including gamma-aminobutyric acid (GABA), flavonoids, proteins, minerals, pigments, vitamins (A, B1, C, and E), dietary fiber, polysaccharides, alkaloids, and polyphenols [4]. Recent reports discussing the broad therapeutic roles of functional ingredients or derived components of barley suggest that it may be the best fit in the modern human diet as a functional food [5,6,7]. Barley saponarin has several health benefits, including anti-inflammatory response [4,8], prevention of bacterial infections [9], regulation of glucose homeostasis, insulin sensitivity [10], reducing low-density lipoprotein (LDL) cholesterol [4], and anti-carcinogenic responses [11]. Similarly, isoorientin from wheat acts as an anti-cancer compound [12] and also possesses anti-inflammatory, antibacterial, antiviral, antiplatelet [13], and antioxidant activities [14]. Wheat (or its derived products) also possesses several beneficial bioactive molecules, including pelargonidin and cyanidin derivatives [15], essential amino acids, fatty acids, flavonoids (e.g., rutin, quercetin, and catechin), vitamin C [16], and policosanols [17]. The *C*-glycosylflavone and policosanol content in barley vary with growth duration [18]. Reports have claimed that sprouts produce higher concentrations of health-promoting molecules than grains [19].

Sprouting implies a series of active biochemical, metabolic, and physiological processes, resulting in the release of active nutrients (e.g., free amino acids and lipid catabolism) for growing plant tissues [18,19]. These metabolites often possess potential health benefits for humans [18,20]. Thus, sprouting is considered one of the easiest natural strategies to enhance nutritional profiles with healthy attributes [20]. Owing to their nutritive values, sprouting seeds has recently received growing interest. Meanwhile, researchers have attempted to identify the presence of novel functional ingredients of sprouts under varying growth and environmental conditions [18,19]. Plants increase the production of a variety of metabolites, in order to mitigate the effects of adverse environmental factors, such as drought [21], salinity [22], high-intensity light or artificial lighting [23], temperature [24], and elevated CO_2_ levels [25]. Therefore, effective management and/or the controlled application of physical energy forms (e.g., light, temperature, and water) may serve as a viable option to enhance the accumulation of health-promoting compounds in sprouts, which has been shown to be successful in previous attempts [25,26,27]. In terms of physical energy forms, light irradiation has been employed in different sprouting seeds, in order to increase metabolites with health-promoting benefits [23,28]. The availability of artificial lighting resources (e.g., light-emitting diodes (LEDs)) renders the possibility of studying the effects of specific light on the concentrations of biologically important metabolites in plants. Herein, the potential effects of a fluorescent lamp (FL) and different spectra of LED light irradiations (white, blue, and red) on beneficial metabolite content were investigated in barley and wheat sprouts. Additionally, we attempted to identify and profile the expression patterns of metabolite biosynthesis-related genes of barley sprouts. This study facilitates understanding of the differential responses relating to *C*-glycosylflavones and policosanols in wheat and barley sprouts.

## 2. Results

### 2.1. Changes of C-glycosylflavone Content in Barley and Wheat Seedlings Exposed to Differential LED Light Irradiation

**Figure 1:**
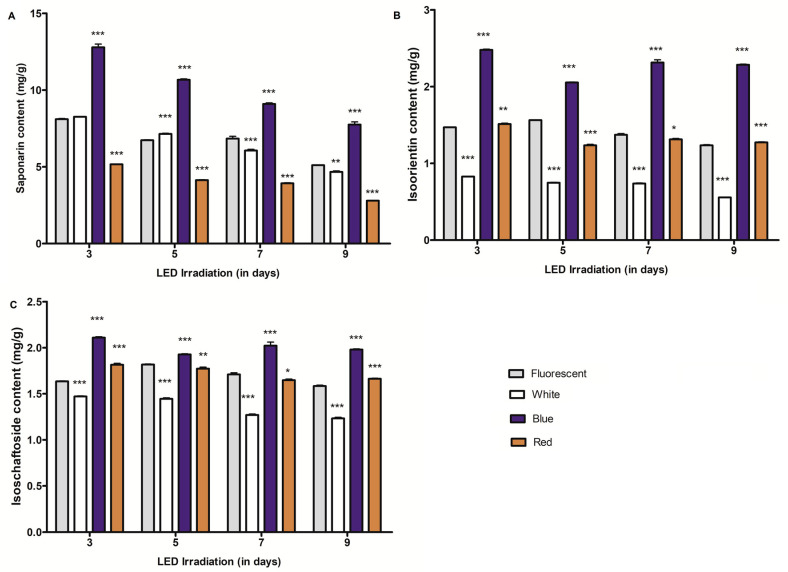
*C*-glycosylflavone content in young barley and wheat seedlings subjected to differential light qualities. (**A**) represents the saponarin content of barley sprouts (mg/g dry weight (DW)) under different light and growth periods, while (**B**,**C**) represent the isoorientin and isoschaftoside contents (mg/g DW), respectively, of wheat sprouts. * (*p* < 0.05), ** (*p* < 0.001), and *** (*p* < 0.0001) indicate the statistical significance.

The changes in saponarin (barley), isoorientin, and isoschaftoside (wheat) content in sprouts treated with different light qualities (FL and white, blue, or red LED irradiation) were measured using ultra-high-performance liquid chromatography (UHPLC) in plant materials harvested after 3–9 days of treatment. The UHPLC results of barley and wheat sprouts revealed that blue LED light irradiation increased the *C*-glycosylflavone content more than other light conditions. The LED irradiation differentially influenced the saponarin content in the barley sprouts. In comparison with FL, LED light irradiation significantly altered the content: Blue light irradiation prominently improved the saponarin content (51.7%–57.7%) across all growth stages and irradiation periods (Figure 1A). The highest concentration of saponarin was observed in sprouts after 3 days of blue LED light irradiation. Interestingly, sprouts treated with red LED light irradiation demonstrated a significant reduction in saponarin content, compared to their respective controls, after 3, 5, 7, and 9 days of irradiation. Conversely, white LED irradiation for three consecutive days did not alter the content; however, on day 5, it statistically significantly increased the contents. The extension of white LED treatment for 7 or 9 days resulted in a reduction of saponarin content. Moreover, regardless of the lighting resource or quality, a consistent reduction in saponarin content was observed in the growing sprouts. Among the growth times and light qualities tested in this study, the highest content of saponarin was observed in blue LED irradiated sprouts on the 3rd day, while red LED radiation remarkably reduced the content in all treatments and sprout growth periods.

Isoschaftoside and isoorientin are the major flavone-*C*-glycosides (*C*-glycosylflavones) frequently reported in wheat and its derived products [29]. In this study, we found that these metabolites were significantly altered in wheat sprouts exposed to LED light irradiation over 3–9 days (Figure 1B,C). LED light irradiation significantly altered the metabolite concentration in barley and wheat sprouts, compared to their content in sprouts treated with traditional fluorescent lamp light conditions. Blue LED light irradiation markedly improved the concentration of isoschaftoside as well as isoorientin, compared to control (FL) or other (white and red) LED treatments. The highest mean concentrations of 2.1 and 2.47 mg (per g dry weight (DW)) of isoschaftoside and isoorientin, respectively, were observed in seedlings subjected to 3 days of blue LED light irradiation. Under similar conditions, the seedlings treated with FL accumulated 1.63 mg and 1.46 mg (per g DW) of isoschaftoside and isoorientin, respectively (Figure 1B,C). On day 3, a slight improvement in isoschaftoside (11.04%) and isoorientin (2.78%) content was also observed in red LED light irradiated sprouts. However, 5 and 7 days of red LED light irradiation significantly reduced the isoorientin and isoschaftoside contents in sprouts. Compared to FL, a maximum of 21.15% reduction in isoorientin content was observed after five days of red LED light irradiation. Under similar conditions, a 2.25% reduction was noted for isoschaftoside content (Figure 1C). Conversely, 9 days of red light irradiation increased the isoorientin (3.15%) and isoschaftoside (5.06%) contents in sprouts. Compared to FL, white LED light irradiation led to reductions in the isoschaftoside (9.81–22.1%) and isoorientin (43.84–55.3%) levels across all growth times. In terms of sprout growth periods, the highest accumulation of metabolites was observed after 3 days of light treatment (Figure 1 and Figure S1).

### 2.2. Effect of LED Light Irradiation on Major Long-Chain Fatty-Alcohol (Policosanol) Biosynthesis in Barley and Wheat Seedlings

**Figure 2:**
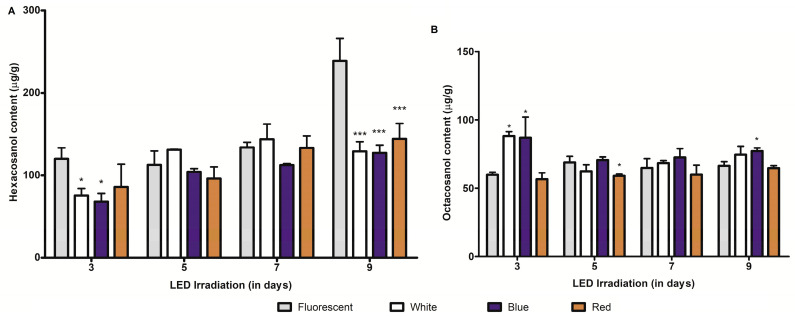
Policosanol content (μg/g DW) in barley and wheat seedlings during different growth periods and light conditions. (**A**) denotes hexacosanol (major policosanol) content in barley sprouts, whereas (**B**) denotes octacosanol content in wheat sprouts. * (*p* < 0.05), and *** (*p* < 0.0001) indicate the statistical significance.

Crude extracts comprised of policosanols from barley and wheat sprouts grown under FL, white, blue, and red LED light irradiations for 3–9 days were profiled and quantified by gas chromatography coupled with mass spectroscopy (GC/MS). GC/MS results of policosanol content in barley and wheat sprouts indicated that growth time was an influential factor in determining the hexacosanol and octacosanol content in these sprouts. Hexacosanol, a major constituent of the policosanol profile of barley seedlings, was altered during LED light irradiation in accordance with sprout growth periods. In general, the hexacosanol content (μg/g DW) in LED light treated samples was significantly lower than that in seedlings treated for 3 or 9 days with FL (Figure 2A). The comparative analysis of hexacosanol content between FL and LED irradiation at the third day of treatment showed that hexacosanol content was significantly reduced for white and blue LED light irradiations. Under these conditions, a maximum reduction of 43.3% was noted for blue LED light irradiation, while white light treatment showed a reduction of 36.9% in hexacosanol content. Conversely, on days 5 and 7, white and blue LED light irradiation did not alter the hexacosanol concentration significantly, in comparison with that of control sprouts. Altogether, the results indicated that the changes in hexacosanol content were not consistent with light qualities, indicating the light quality is not the only factor influencing changes in hexacosanol biosynthesis. Interestingly, hexacosanol content in sprouts treated with either white or blue LED irradiation for 5, 7, and 9 days was higher than the hexacosanol content in sprouts treated only for three days under similar conditions. Likewise, the octacosanol content (μg/g DW) in wheat seedlings was analyzed using GC/MS (Figure S2). The results showed that the octacosanol content was altered irregularly by different LED light qualities (Figure 2B). A comparative analysis of FL and LED treatments indicated that blue LED light irradiation for 3 and 9 days enhanced the octacosanol content in wheat sprouts. In comparison with FL, white light irradiation also increased the content in sprouts exclusively on day 3. Conversely, red LED irradiation resulted in octacosanol content reduction (on day 5). Nonetheless, the magnitude of change in content was not consistent for any of the LED treatments.

### 2.3. Effect of Growth Periods on C-glycosylflavones and Policosanol Content in Young Barley and Wheat Seedlings

To understand the effect of sprout growth periods (3–9 days) on the metabolite content (saponarin, isoorientin, isoschaftoside, hexacosanol, and octacosanol) under fluorescent and LED light irradiation, we conducted a further investigation. Of all the growth times, the saponarin content of barley and isoorientin/isoschaftoside contents of wheat seedlings were found to be the highest on the 3rd day of the treatment (Figure S3A–C). The contents of these *C*-glycosylflavones showed a declining trend with an increase in growth times (i.e., after 5, 7, and 9 days) in barley and wheat seedlings (Figure S3A–C). However, the magnitude of reduction in metabolite content varied with different light qualities and metabolites in these sprouts. The saponarin content of barley was reduced by 37.96%, 43.58%, 39.43%, and 45.93% for FL, white, blue, and red LED light irradiation, respectively, after the further growth period of 9 days. A statistically significant negative correlation (−0.93 to −0.99) between saponarin content and sprout growth period was observed during FL and LED light (white, blue, and red) irradiation (Table 1). A similar attempt to establish relationships between isoorientin content and growth periods in wheat sprouts under FL and LED treatment showed that isoorientin accumulation was negatively correlated with growth periods under specific light (FL, white, and red) treatments. Furthermore, isoschaftoside accumulation is negatively regulated under white and red LED light treatments across growth periods. Nonetheless, their relationships in blue LED irradiated sprouts were inconclusive.

In contrast to *C*-glycosylflavone content, the hexacosanol content in barley sprouts gradually increased with the extension of the growth period. In fact, the highest accumulation of hexacosanol was observed in barley sprouts after 9 days of FL and LED light (white, blue, and red) irradiation treatments (Figure S3D). The correlation coefficient (Pearson) analysis showed that positive correlations (0.69 to 0.91) existed between hexacosanol content and growth period, which was statistically significant in both FL and LED irradiated seedlings (Table 1). Unlike hexacosanol, the positive relationship between octacosanol content and wheat growth period was not clear (Figure S3E). As indicated in Figure S3E, the growth period did not impose any significant effect on octacosanol content under white, red, and blue LED or FL light irradiation. Nonetheless, a comparison of growth periods under white LED irradiation showed significant differences in octacosanol concentration in wheat sprouts.

### 2.4. Expression Profiling of Potential Genes Involved in the Biosynthesis of C-glycosylflavones/Flavonoids and Policosanols in Barley Seedlings under Different Light Conditions and Growth Times

**Figure 3:**
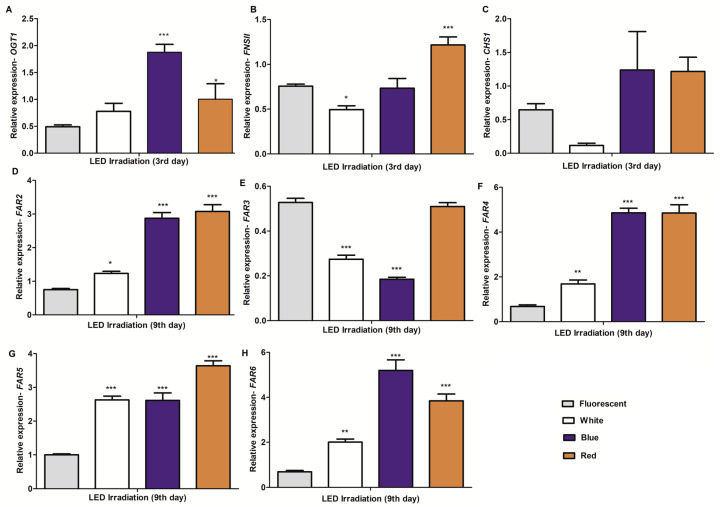
Relative quantification of expression changes in flavonoid and policosanol biosynthesis-related genes in barley sprouts. (**A**–**C**) represent the relative expression levels of *UDP-Glc: Isovitexin 7*-*O-glucosyltransferase 1* (*OGT1*), *flavone synthase II* (*FNSII*), and *chalcone synthase 1* (*CHS1*), respectively. Likewise, (**D**–**H**) represent the expression patterns of different classes of *fatty acyl-coenzyme A reductase (FAR)* genes. The results represent the qRT-PCR-based relative quantification of genes in barley sprouts exposed to fluorescent and LED (white, blue, and red) light irradiations. The gene expression was normalized using the internal control *HvActin*. * (*p* < 0.05), ** (*p* < 0.001), and *** (*p* < 0.0001) indicate the statistical significance.

The homology-based gene identification approach was used to predict the potential genes associated with the biosynthesis of flavones/flavonoids and long-chain fatty alcohols in the barley genome. The mRNA sequences of potential candidate genes were designated according to their highest sequence similarity to known genes of other crops (Table S1). As shown in Figure S3, sprout growth periods were one of the factors that greatly influenced the saponarin and hexacosanol contents; thus, we selected the growth periods associated with high accumulation of these metabolites for investigation of expression analyses of flavonoid and fatty alcohol biosynthesis-related genes. The expression levels of *HvCHS1*, *HvFNSII,* and *HvOGT1* for flavonoid biosynthesis and *HvFAR2*, *HvFAR3*, *HvFAR4*, *HvFAR5,* and *HvFAR6* for policosanol biosynthesis were significantly altered under FL, white, blue, and red light irradiation (Figure 3A–H). The expression pattern of *HvOGT1* under differential light conditions was positively correlated (0.68) with saponarin content in barley (Table 1). In terms of light responses, the expression of *FNSII* was significantly upregulated in red LED irradiated seedlings, while its expression was downregulated by white LED light, in comparison with FL or blue LED light. The light-responsive expression of *CHS1* was slightly changed under LED irradiation, but the changes were negligible (Figure 3C). In terms of policosanol biosynthesis-related gene expression, the expression pattern of *HvFAR3* had a positive correspondence (0.67) with hexacosanol accumulation (Table 1). The *HvFAR3* expression was reduced under white and blue LED light irradiation, while it was unaltered in red LED light irradiated seedlings. Interestingly, the expression patterns of *HvFAR2, HvFAR4, HvFAR5,* and *HvFAR6* were negatively correlated (−0.59 to −0.76) with hexacosanol content in barley sprouts. Their expressions were upregulated by one or more LED light irradiations. *HvFAR2* and *HvFAR5* showed their highest expression under red LED light irradiation (Figure 3D,F–H). Likewise, the highest expression of *HvFAR6* was observed under blue LED irradiation. It is clear that *HvFAR3* is possibly involved in hexacosanol biosynthesis in barley sprouts.

### 2.5. Influence of LED-Light Irradiation on Seedling Growth

**Figure 4:**
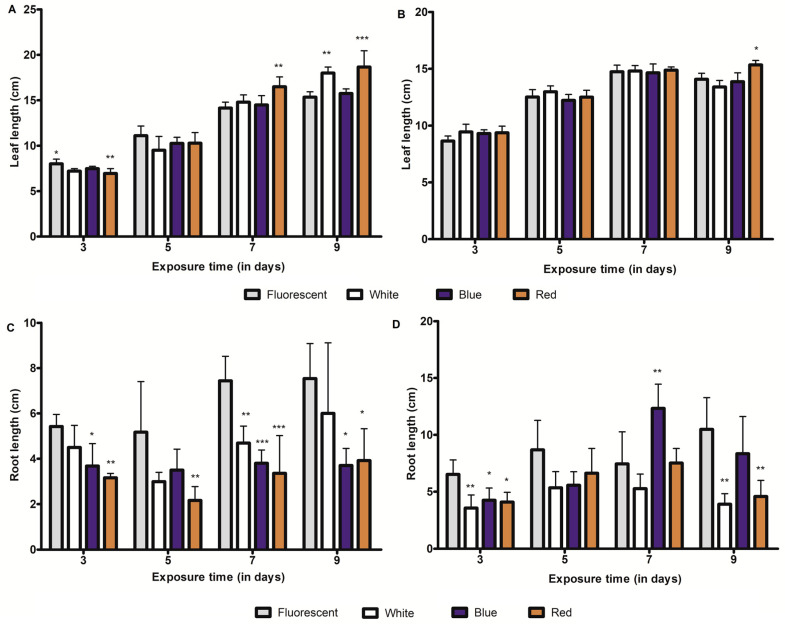
Leaf and root growth parameters of fluorescent and light-emitting diode (LED) light (white, blue, and red) irradiated barley and wheat seedlings at different growth periods. (**A**,**C**) represent barley growth parameters, while (**B**,**D**) represent the growth parameters of wheat sprouts. * (*p* < 0.05), ** (*p* < 0.001), and *** (*p* < 0.0001) indicate the statistical significance.

In most of the LED treatments in wheat seedlings, there were no statistically significant differences in leaf length, compared to control (FL) seedlings (Figure 4). However, the root growth of wheat sprouts treated with LED light irradiation for 3 days was significantly reduced, compared to that of the controls. Interestingly, further LED treatment on 5 and 7 days did not produce a significant impact on root growth, except under blue LED irradiation on day 7. Nonetheless, continuing white and red LED irradiation up to 9 days reduced root growth, which was statistically significant. In terms of light quality, the highest reduction in root growth was observed under white LED irradiation. Like wheat sprouts, the root growth in barley sprouts was altered by specific light qualities, in accordance with the growth period. Red LED irradiation consistently reduced barley root growth across all growth periods. White or blue LED irradiation also resulted in root growth reduction in one or more growth periods. Although initial treatment with red LED irradiation (on day 3) reduced leaf growth in barley sprouts, the extending the irradiation for 7 or 9 days had a positive impact on barley leaf growth. LED treatment in wheat sprouts showed that the leaf growth was mostly not altered.

## 3. Discussion

Phytochromes and cryptochromes are the specialized photoreceptors of plants that sense the spectral quality and quantity, transducing the light signal to regulate genes responsible for secondary metabolite production [30]. Therefore, it is possible to determine the metabolic composition or to enhance the nutritional functionality of the target crops through selective application of light resources and photoperiodism. The application of LEDs for special metabolite production is considered promising, where it has been shown that the metabolic profiles also depend on several other factors, including crop genetics [18,26,31]. The increasing application of LED irradiation sources for the development of designer foods/functional foods may revolutionize the food industry. In this study, we attempted to enhance the *C*-glycosylated flavones/flavonoids and policosanol contents in barley and wheat sprouts using varying light qualities.

*C*-glycosylated flavones constitute the major portion of flavonoids found in barley seedlings [32]. Saponarin (isovitexin-7-O-glucoside) is a major *C*-glycosylated flavone, which is naturally present in young barley seedlings [11]. Among cellular organelles, saponarin is efficiently stored in vacuoles [33] and high accumulation is typically observed in primary leaves [4]. Similarly, isoorientin and isoschaftoside are the major *C*-glycosylated flavones often reported in wheat seedlings [29]. These *C*-glycosylflavones have potential roles as beneficial flavonoids in the human diet [4,8,34]. In this study, we found that the *C*-glycosylflavone (saponarin, isoorientin, and isoschaftoside) content was high in the early growth stages of seedlings, where the maximum accumulation was induced by blue LED light irradiation. We found an inverse relationship between *C*-glycosylated flavone content and growth times, indicating that the *C*-glycosylflavone content remains high in young sprouts. In a previous study investigating barley sprouts (13–56 days post-sprouting), it was stated that the saponarin content continued to decline with increasing growth periods [9]. Now, it is clear that the saponarin content in barley sprouts starts declining just three days post-sprouting. In terms of light quality, blue LED light had a positive impact on saponarin content. Blue LED light showed the highest accumulation (57.7% and 68.68% than that in the control, respectively) in barley and wheat sprouts. On the other hand, the impact of red LED light irradiation on saponarin content in barley, and isoorientin and isoschaftoside in wheat sprouts, seemed to differ. In barley, the saponarin content was reduced, while the levels of isoorientin and isoschaftoside were increased in wheat sprouts, suggesting that the effect of red LED light is specific to metabolites and/or crops.

Policosanol (PC) is another beneficial metabolite in the human diet, which is frequently found in cuticular waxes in primary leaves of young cereal sprouts. It represents a mixture of long-chain fatty alcohols (20–36 carbon) mostly comprised of docosanol (C22), tetracosanol (C24), hexacosanol (C26), octacosanol (C28), and triacontanol (C30) [35,36]. Octacosanol and policosanol (long-chain saturated fatty alcohols) are useful in preventing high-fat diet-induced obesity [37]. Owing to its importance in lowering blood cholesterol and protection from platelet aggregation, it has been commercialized in the health industry for a long time [35]. Among the PCs, hexacosanol (C26) and octacosanol (C28) are often observed in barley and wheat sprouts [36,38]. LED light irradiation showed a differential influence on the policosanol content in barley and wheat sprouts, suggesting that the LED response is likely specific to either metabolites or crops. Compared to FL conditions, LED light irradiation reduced the hexacosanol content in barley, while a similar condition in wheat sprouts showed an irregular pattern of octacosanol accumulation; suggesting that factors other than light quality also influence its content in wheat sprouts. Interestingly, in most cases, the policosanol content in barley and wheat sprouts gradually increased with the extension of growth time. Statistical analyses confirmed that the saponarin content under LED treatment was negatively correlated with barley growth periods. A similar negative relationship between content and growth period was also evident in wheat sprouts, albeit restricted to white and/or red LED light treatments. Unlike *C*-glycosylflavone, the hexacosanol content in sprouts appeared to have a positive correlation with sprout growth periods, suggesting the importance of growth level in the determination of policosanol content in barley. Our study corroborates previous findings which have reported a positive correspondence between hexacosanol and growth periods in barley [36]. However, a similar correlation pattern was not observed between octacosanol content and growth periods in FL, white, and blue LED irradiated wheat sprouts, possibly suggesting that this relationship might be specific to crop genetics. Altogether, our results show that light qualities and growth periods are two crucial factors in determining the C-glycosylflavone and policosanol contents in barley and wheat sprouts. In addition, light conditions are important parameters in determining the photomorphogenesis of plants [39]. Herein, we found that red LED light irradiation mostly reduced the root growth of sprouts, while white and blue LED light mediated root growth inconsistently across growth periods, indicating the role of other factors regulating growth parameters. The LED light responses of leaves of young seedlings also indicated that LED irradiation does not induce a regular growth pattern for leaves in barley and wheat sprouts. At this stage, it is difficult to conclude the growth impact of LED light irradiation, as this study utilized low intensity LED spectra. Further studies concerning the selection of optimum light intensity and LED spectra for enhancing sprout growth are, therefore, essential.

An interesting observation is that comparison of the metabolite accumulation trends revealed the *C*-glycosylflavones and policosanol contents to have an inverse relationship in barley sprouts. It is unknown whether this is due to a balancing act of metabolic pathways or just an influencing act of sprout growth periods. It warrants further in-depth studies, in order to understand the underlying mechanisms of metabolite biosynthesis. The molecular basis for the specialized metabolite accumulation to a particular physiological condition is mainly due to changes in the expression pattern of one or more specialized biosynthetic genes. Lee et al. [11] suggested that the expression pattern of UDP-Glc: Isovitexin 7-O-glucosyltransferase (OGT) is likely responsible for saponarin biosynthesis in barley. *OGT* is responsible for the conversion of isovitexin to saponarin in barley [33]. There are two classes of OGT (*OGT1* and *OGT2*) found in the barley genome, where a change in the expression level of *OGT1* has been associated with saponarin concentration [11]. In the present study, we found that a positive correlation exists between the *HvOGT1* expression pattern and saponarin accumulation, suggesting the possibility of *OGT1* involvement in saponarin biosynthesis. Both metabolite accumulation and *HvOGT1* expression were found to be highest after 3 days of treatment. It is clear that blue LED light irradiation accumulated saponarin by upregulating *HvOGT1* expression in barley sprouts. The flavonoid biosynthesis pathway gene, *HvCHS1*, is not linked with saponarin accumulation, indicating the possibility of a specific pathway controlling saponarin biosynthesis in barley. Studies on other crops have shown that fatty acyl-coenzyme A reductases (FAR) are involved in long-chain primary alcohol biosynthesis, part of the cuticular waxes found in leaf surfaces [40]. Studies have also shown that the number of *FAR* genes and their functions may vary, according to species-specific genetics. In other crops, it is evident that *FAR2* and *FAR3* are responsible for the biosynthesis of C_26_ and C_28_ primary alcohols [41]. Another report has claimed that at least five *FARs* are responsible for primary alcohol (C_16_ to C_28_) biosynthesis in *Aegilops tauschii* [42]. Until recently, there has been no information about *HvFARs* and their potential role in hexacosanol biosynthesis. Identification of the *FARs* responsible for policosanol biosynthesis is inevitable for tailoring metabolic pathways towards enhanced production. Hence, we used the homology-based gene identification method to predict the gene sequences of *HvFAR2, HvFAR3, HvFAR4, HvFAR5*, and *HvFAR6* from available barley genome information. We also measured their expression changes using quantitative PCR during differential light treatments and growth periods, in order to infer their relationship to hexacosanol biosynthesis. Of all the *HvFARs* analyzed in this study, the expression changes of *HvFAR3* were positively associated with hexacosanol accumulation, suggesting their involvement in hexacosanol biosynthesis. Other *HvFARs* did not have a positive correlation with hexacosanol content, suggesting that they may not be associated with its biosynthesis. In this study, we identified the potential candidate genes involved in saponarin and hexacosanol biosynthesis. These genes can be effectively used to enhance the metabolite concentration by means of genetic manipulation. We also provided an expression atlas of *HvFARs* during LED light irradiation treatment in sprouts, which may be useful in future studies associated with other policosanol biosynthesis routes. A system-wide identification and characterization would add more information about the genetic factors controlling metabolite biosynthesis in barley sprouts.

To conclude, we showed that light qualities and growth times are crucial factors determining the contents of *C*-glycosylflavones and policosanols in barley and wheat sprouts. Blue LED light may be useful for increasing the *C*-glycosylflavone contents in cereal sprouts. Regardless of the light quality, management of growth time of sprouts is essential for policosanol content. This study will help to maximize the beneficial flavonoids and policosanol contents in cereal sprouts through LED applications in the future.

## 4. Materials and Methods

### 4.1. Plant Materials, Growth Condition, and LED Treatment

**Figure 5:**
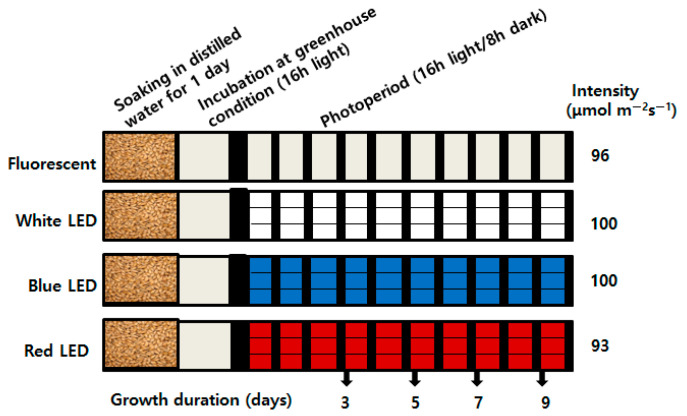
Schematic representation of experimental design used for LED treatment on barley and wheat seedlings. The diagram illustrates the germination method, growth period, and light quality and intensity.

The barley (*Hordeum vulgare* L. variety “Keunalbori No.1”) and wheat (*Triticum asetivum* L. variety “Baekchalmil”) seeds used by this study were sourced from the National Institute of Crop Science (Korea). The seeds at full maturity were separated by soaking thrice in tap water. Then, visually healthy seeds were once again soaked separately in distilled water for one day. For germination, seeds were sown on 16 plastic sprout cultivating trays (90 cm by 30 cm; ~180 g per tray) and maintained in greenhouse conditions (25 ℃, 16 h photoperiod) for one day. Four trays with sprouting seeds (per treatment) were moved to individual growth chambers (Mokmin Co., Ltd, Suwon, South Korea) equipped with FL or different spectra LED light irradiations (470 nm for blue, 380 nm for white, and 660 nm for red) over 9 days (Figure 5). Sprouts were watered at regular intervals. The photosynthetic photon flux density (PPFD) of FL (96 μmol m^−2^s^−1^), blue (100 μmol m^−2^s^−1^), white (100 μmol m^−2^s^−1^), and red (93 μmol m^−2^s^−1^) lights was measured at plant level by using a quantum meter (Apogee Instruments, Logan, USA). Fresh leaves and coleoptiles of each treatment were harvested simultaneously at 3, 5, 7, and 9 days after irradiation (DAI). All samples were frozen in liquid nitrogen and stored at −80 ℃ for RNA extraction and metabolomic analyses. All sprouting seeds throughout the treatments were maintained in a growth chamber with 16 h photoperiods, 60–80% humidity, and 22–25 ℃.

### 4.2. Extraction of C-glycosylated Flavones and Measurement

The extraction method of Lee et al. [11] was used for the preparation of crude extracts from dried barley and wheat seedlings. An ultra-high-performance liquid chromatograph equipped with UV detectors (Dionex Ultimate 3000; Thermo Scientific, Waltham, MA, USA) and reversed-phase HPLC column (ACQUITY UPLC BEH C18, 2.1 mm by 100 mm) was utilized for separation and quantitative analyses [8]. One gram of freeze-dried and chopped seedlings from each treatment was extracted by treating with either 50% ethanol (barley) or 100% methanol (wheat) on a shaker for 24 h at room temperature. The ethanolic or methanolic extracts were then filtered through 0.2 μm syringe filters. Following evaporation under vacuum, the extracts were dissolved in 10% dimethyl sulfoxide (DMSO) containing 50% ethanol and 1.3 μL was injected into the column for separation and detection at 325 nm. The mobile phase was comprised of trifluoroacetic acid (TFA) (0.1%) in water (A) and acetonitrile (B) with a flow rate of 0.5 mL/min, which was applied for separation of the analyte. Saponarin, isoorientin, and isoschaftoside molecules were identified by comparing the retention times to those of standards (obtained from Extrasynthese, Lyon, France and NICS, Jeonju, Korea). A standard calibration curve was prepared by plotting the peak area (y) of the chromatogram and the respective concentrations (31.25, 62.5, 125, 250, 500, and 1000 μg/mL) (x). The equations of the calibration curves for saponarin, isoorientin, and isoschaftoside were y = 0.0876x + 0.3514 (r^2^ = 0.999), y = 0.0917x −0.5536 (r^2^ = 0.998), and y = 0.0.0547x + 0.22935 (r^2^ = 0.999), respectively. The metabolite content (mg/g DW) from three technical replicates of biologically independent samples were used as input for the statistical analyses.

### 4.3. Extraction and Quantification of Policosanols from Barley and Wheat Seedlings

The preparation of barley and wheat crude extracts, policosanol standards, GC/MS parameters, and the quantification method of GC/MS as described elsewhere [17,36], was used in this study. Briefly, 1 g of freeze-dried and chopped samples collected from all treatments was extracted separately into 10 mL of hexane on a shaker for 24 h at room temperature. The supernatant of the mixture was collected by centrifugation at 3000 g for 3 min, filtered through a syringe filter with a pore size of 0.45 μm (Whatman Inc., Maidstone, UK), and kept under vacuum conditions until the hexane was completely removed. To the final extract, 250 μL of N-Methyl-N-trimethylsilyfluoroacetamide (MSTFA) and 0.5 mL of chloroform was added and stirred for 15 min at 60 °C. Chloroform was added to make up one ml of sample and, then, one μL was injected into the gas chromatograph using an auto sampler with a split ratio of 1:5. The GC was equipped with an HP-5MS UI (diphenyl 5%-dimethylsiloxane 95% co-polymer) capillary column (30 m by 0.25 μm by 0.25 μm film thickness) and a 5977A series mass spectroscopy (Agilent Technologies, Palo Alto, CA). The policosanol content was quantified according to the methods of Ra et al. [17]. The policosanol standards—Eicosanol (C_20_), heneicosanol (C_21_), docosanol (C_22_), tricosanol (C_23_), tetracosanol (C_24_), hexacosanol (C_26_), heptacosanol (C_27_), octacosanol (C_28_), and triacontanol (C_30_)—hexane, and chloroform solutions were purchased from Sigma (Sigma-Aldrich, St. Louis, MO).

### 4.4. Identification of Saponarin and Policosanol Biosynthetic-Related Genes

In general, flavone/flavonoid and long-chain fatty alcohol (or primary alcohol) biosynthesis pathways comprised of one or more enzymes and their information in barley are limited. Hence, the gene names and sequence information of other crops were searched for sequence homologies to unearth orthologous transcripts (full and partial) in *Hordeum vulgare* and *Triticum asetivum* species. The gene sequences were retrieved from NCBI Gene [43], PlantsDB [44], and GrainGenes [45] databases. For annotation, potential orthologous gene sequences were searched for the presence of conserved domains using the NCBI Conserved Domains [46] database. Transcripts encoding for flavone/flavonoid-related—UDP-Glc: *OGT1*, flavone synthase II (*FNSII*), and chalcone synthase 1 (*CHS1*)—and fatty alcohol-related—fatty acyl-CoA reductase 2 (*FAR2*), *FAR3*, *FAR4*, *FAR5*, and *FAR6*— biosynthesis were identified in barley. The designated gene sequences are listed in Table S1, which were utilized in primer synthesis for qRT-PCR-based gene expression profiling.

### 4.5. Analysis of Expression Pattern of Biosynthetic Related Genes in Seedlings Subjected to LED Light Irradiation

Approximately 100 mg of powdered leaf samples of five or more seedlings were utilized for total RNA extraction using an RNeasy Plant Mini kit (Qiagen, Hilden, Germany). First-strand cDNA was synthesized from DNA decontaminated total RNA (5 μg) of each treatment using amfiRivert cDNA Synthesis Platinum Master mix (GenDepot, Katy, TX, USA). The diluted cDNA (1:10) was used as a template, along with gene-specific primers (Table S1) and AccuPower 2× GreenStar Master Mix (Bioneer, Daejeon, Korea), for relative quantification of saponarin, policosanol, and general flavonoid biosynthesis-related genes in quantitative RT-PCR with CFX96^TM^ Real-Time PCR Detection System (Bio-Rad, Hercules, CA, USA). *HvActin* was used as an internal control. The relative quantification of gene expression was calculated using the 2^-ΔΔCT^ method.

### 4.6. Phenotyping of LED-Treated Cereal Sprouts

The effect of LED light irradiation on the growth of wheat and barley sprouts was evaluated from five or more seedlings of each treatment, collected after 3–9 days of treatment. The length of all primary leaves and roots were measured. The mean values of growth parameters were compared between treatments and the mean differences were evaluated by statistical significance analysis.

### 4.7. Statistical Analyses

All treatments and experiments in this study had at least three independent biological and technical replicates. The mean values are presented in graphs drawn using the GraphPad Prism 5 software, while standard deviations are represented as error bars. One-way analysis of variance (one-way ANOVA) and Tukey′s HSD test were carried out to assess the significant variations existing between the treatments and analyses performed in this study. Statistics by ANOVA test are shown; * *p* < 0.05, ** *p* < 0.001, and *** *p* < 0.0001. Pearson correlation coefficients and their significance were measured using the Social Science Statistics [47].

## Figures and Tables

**Table 1:** Analysis of correlation statistics (Pearson correlation) among metabolite content, growth periods, and their respective biosynthesis-related gene expression patterns across different light qualities.

	Metabolite Content vs. Gene Expression							
	*HvOGT1*	*HvFNSII*	*HvCHS1*	*HvFAR2*	*HvFAR3*	*HvFAR4*	*HvFAR5*	*HvFAR6*
Saponarin	0.68 (*p* = 0.01) **	−0.53 (*p* = 0.07)	0.13 (*p* = 0.68)	--	--	--	--	--
Hexacosanol	--	--	--	−0.59 (*p* = 0.04) *	0.67 ( *p*= 0.01) **	−0.62 (*p* = 0.03) *	−0.76 (*p* = 0.004) **	−0.69 (*p* = 0.01) **
Growth periods (–9 days) vs. metabolite content								
	Fluorescent		White		Blue		Red	
Saponarin	−0.93 (*p* = 0.00001) ****		−0.99 (*p* = 0.00001) ****		−0.99 (*p* = 0.00001) ****		−0.97 (*p* = 0.00001) ****	
Isoorientin	−0.82 (*p* = 0.001) ***		−0.92 (*p* = 0.00002) ****		−0.23 (*p* = 0.47)		−0.66 (*p* = 0.01) **	
Isoschaftoside	−0.32 (*p* = 0.31)		−0.94 (*p* = 0.00001) ****		−0.47 (*p* = 0.12)		−0.90 (*p* = 0.00006) ****	
Hexacosanol	0.79 (*p* = 0.001) ***		0.69 (*p* = 0.01) **		0.91 (*p* = 0.00004) ****		0.81 (*p* = 0.001) ***	
Octacosanol	−0.07 (*p* = 0.82)		−0.43 (*p* = 0.16)		−0.17 (*p* = 0.57)		−0.04 (*p* = 0.90)	

* *p* < 0.05, ** *p* < 0.001, *** *p* < 0.0001, **** *p* < 0.00001 indicates statistical significance (replicates were included for measuring Pearson correlation).

## Supplementary Materials

The following are available online at https://www.mdpi.com/2223-7747/9/11/1502/s1, Figure S1: Representative chromatogram of saponarin and hexacosanol of barley seedlings exposed to differential LED light treatments. Figure S2: Chromatogram of isoorientin, isoschaftoside, and octacosanol of wheat seedlings exposed to differential LED light treatments. Figure S3: Effect of growth periods on the C-glycosylflavone and policosanol contents in fluorescent and LED light irradiated barley and wheat sprouts. Table S1: List of genes, respective gene and protein IDs, primer sequences, and their annealing temperatures used in the quantitative RT-PCR assay.
