# Supplementary material for: Changes in Beneficial C-glycosylflavones and Policosanol Content in Wheat and Barley Sprouts Subjected to Differential LED Light Conditions
Source: Plants (Basel). 2020 Nov 6;9(11):1502. doi: 10.3390/plants9111502 (PMC7694615; doi:10.3390/plants9111502)
Supplement: Supplementary file 1 [file plants-09-01502-s001.pdf]

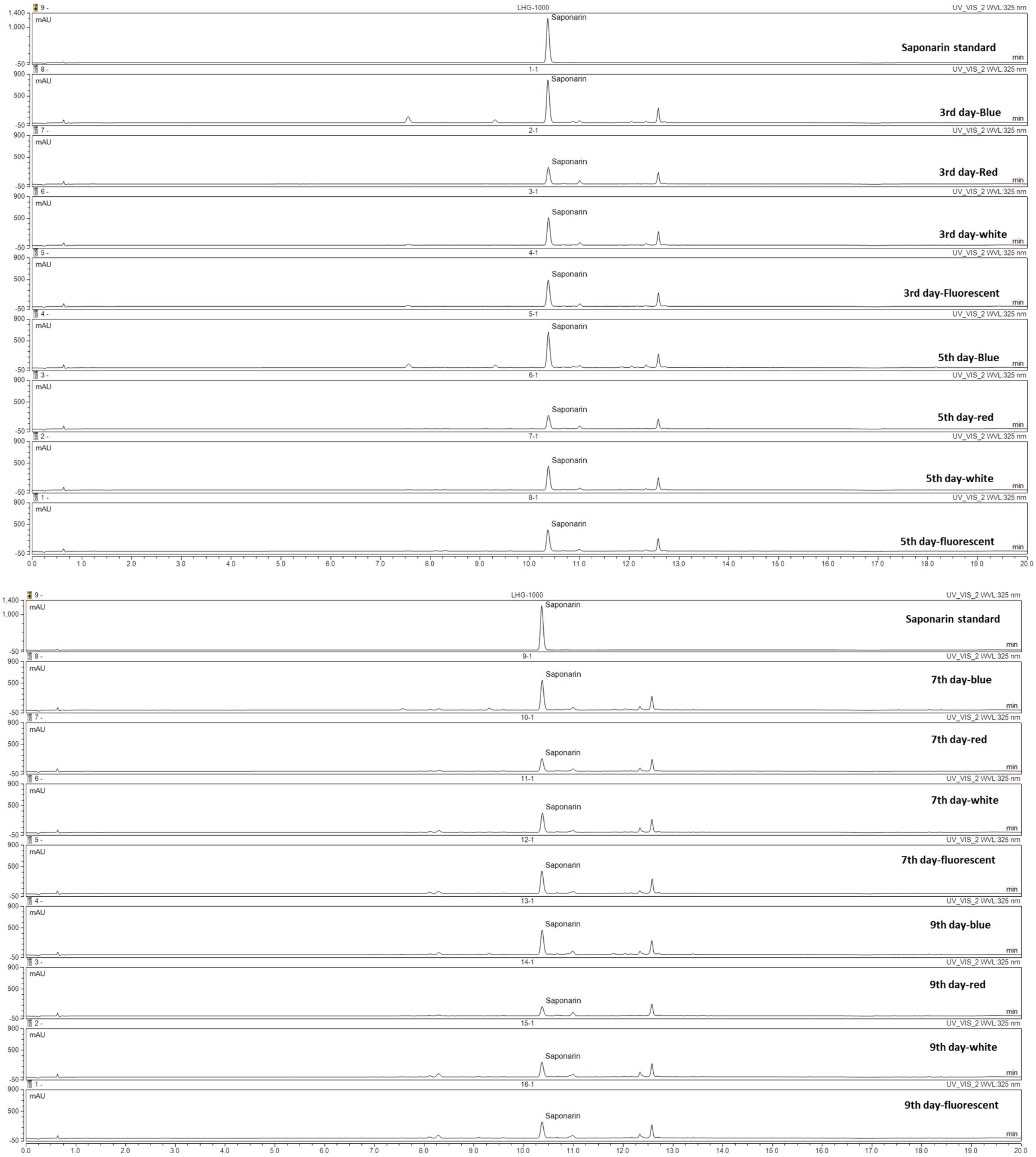

Figure 1. The representative chromatogram of saponarin and the hexacosanol of barley seedlings exposed to differential LED light treatments.

1: Eicosanol, 2: Heneicosanol, 3: Docosanol, 4: Tricosanol, 5: Tetracosanol, **6: Hexacosanol**, 7: Heptacosanol, 8: Octacosanol, 9: Triacontanol

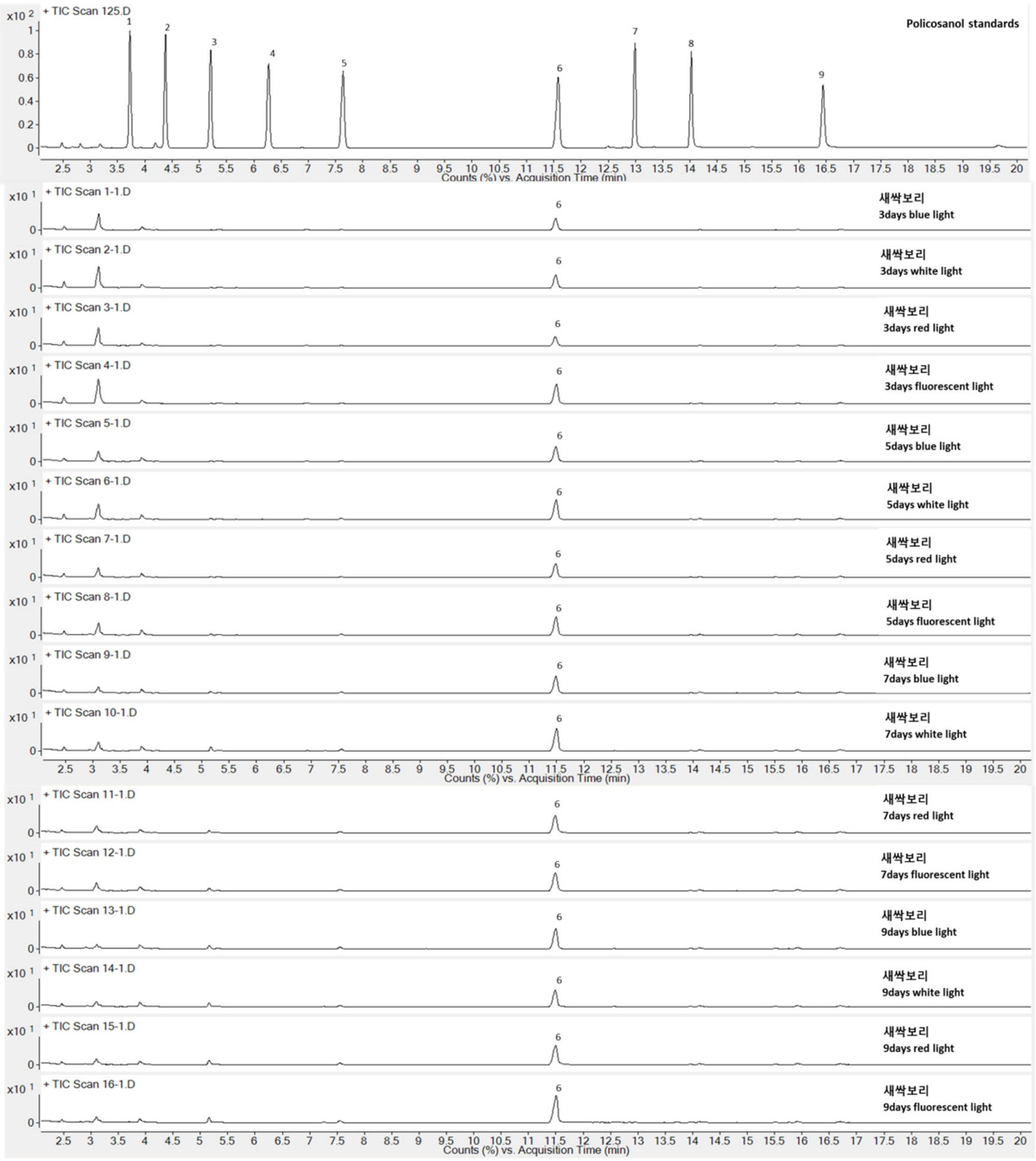

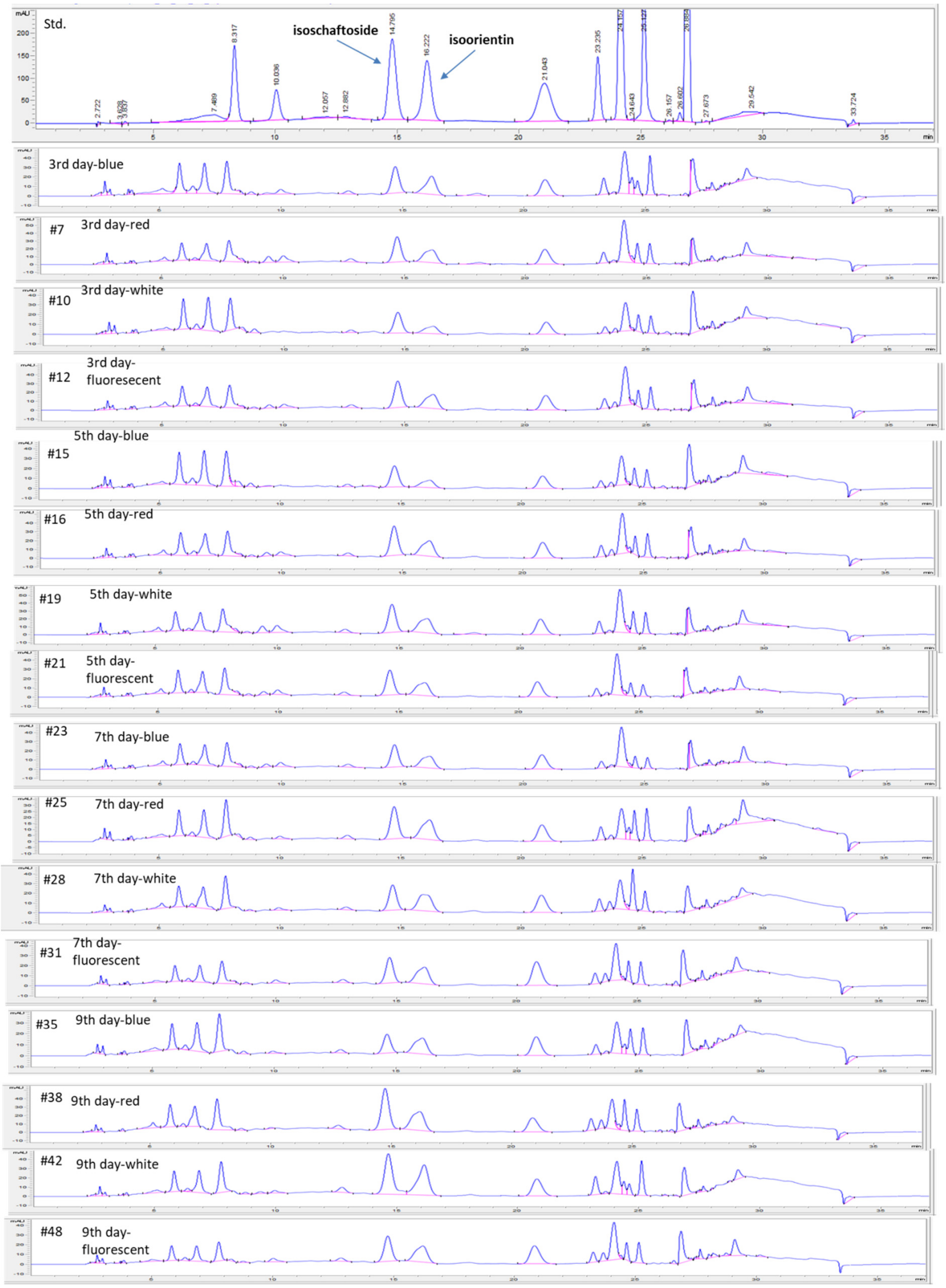

Std.
isoschaftoside
isoorientin
mAU
min
2.722
7.489
8.317
10.036
12.057
12.882
14.795
16.222
21.043
23.235
24.643
26.157
26.602
27.673
29.542
33.724
3rd day-blue
#7 3rd day-red
#10 3rd day-white
#12 3rd day-fluoresecent
5th day-blue
#15
#16 5th day-red
#19 5th day-white
#21 5th day-fluorescent
#23 7th day-blue
#25 7th day-red
#28 7th day-white
#31 7th day-fluorescent
#35 9th day-blue
#38 9th day-red
#42 9th day-white
#48 9th day-fluorescent

1: Eicosanol, 2: Heneicosanol, 3: Docosanol, 4: Tricosanol, 5: Tetracosanol, **6:** Hexacosanol, 7: Heptacosanol, 8: **Octacosanol**, 9: Triacontanol

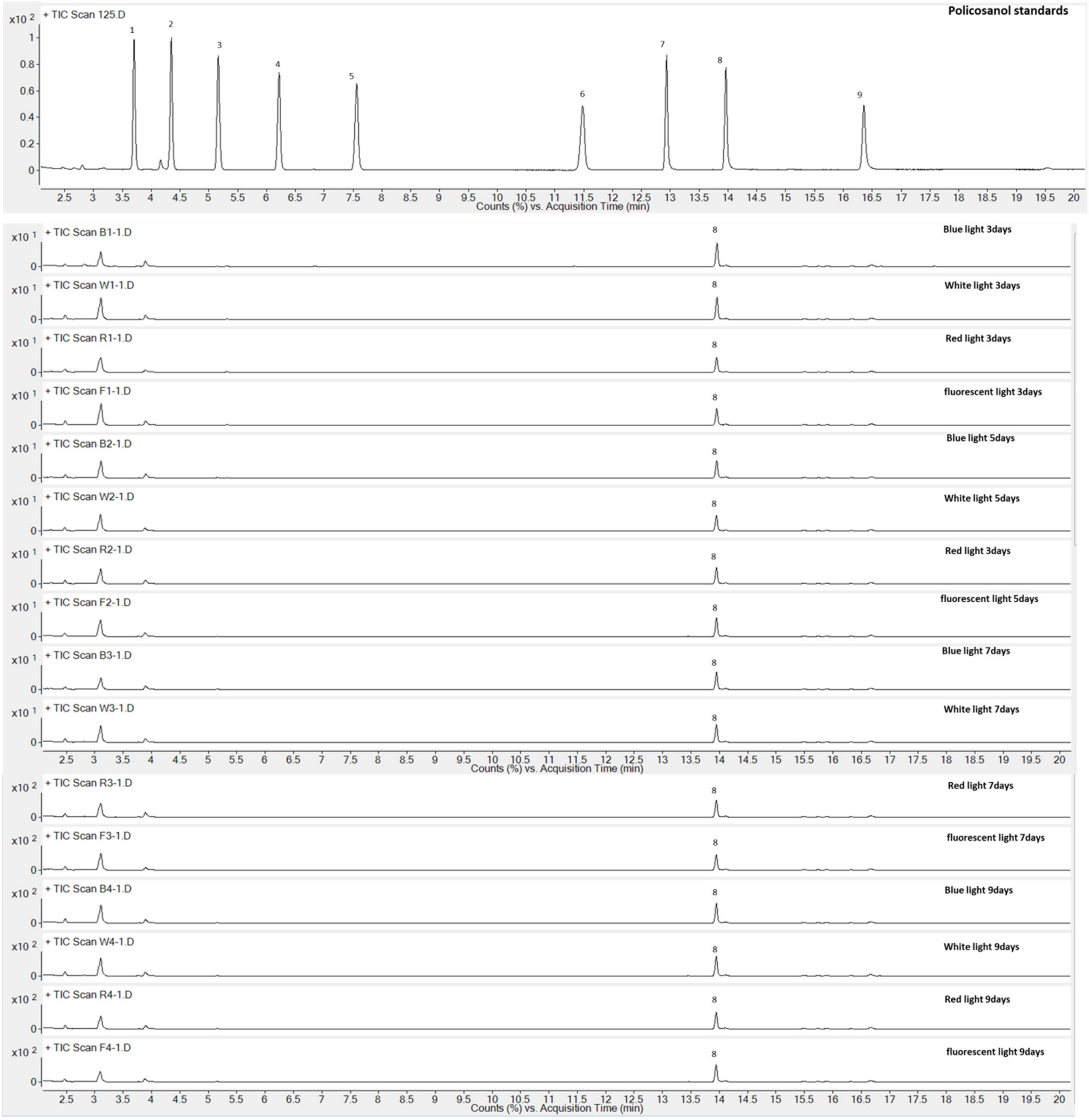

Figure 2. The chromatogram of isoorientin, isoschaftoside and the octacosanol of wheat seedlings exposed to differential LED light treatments.

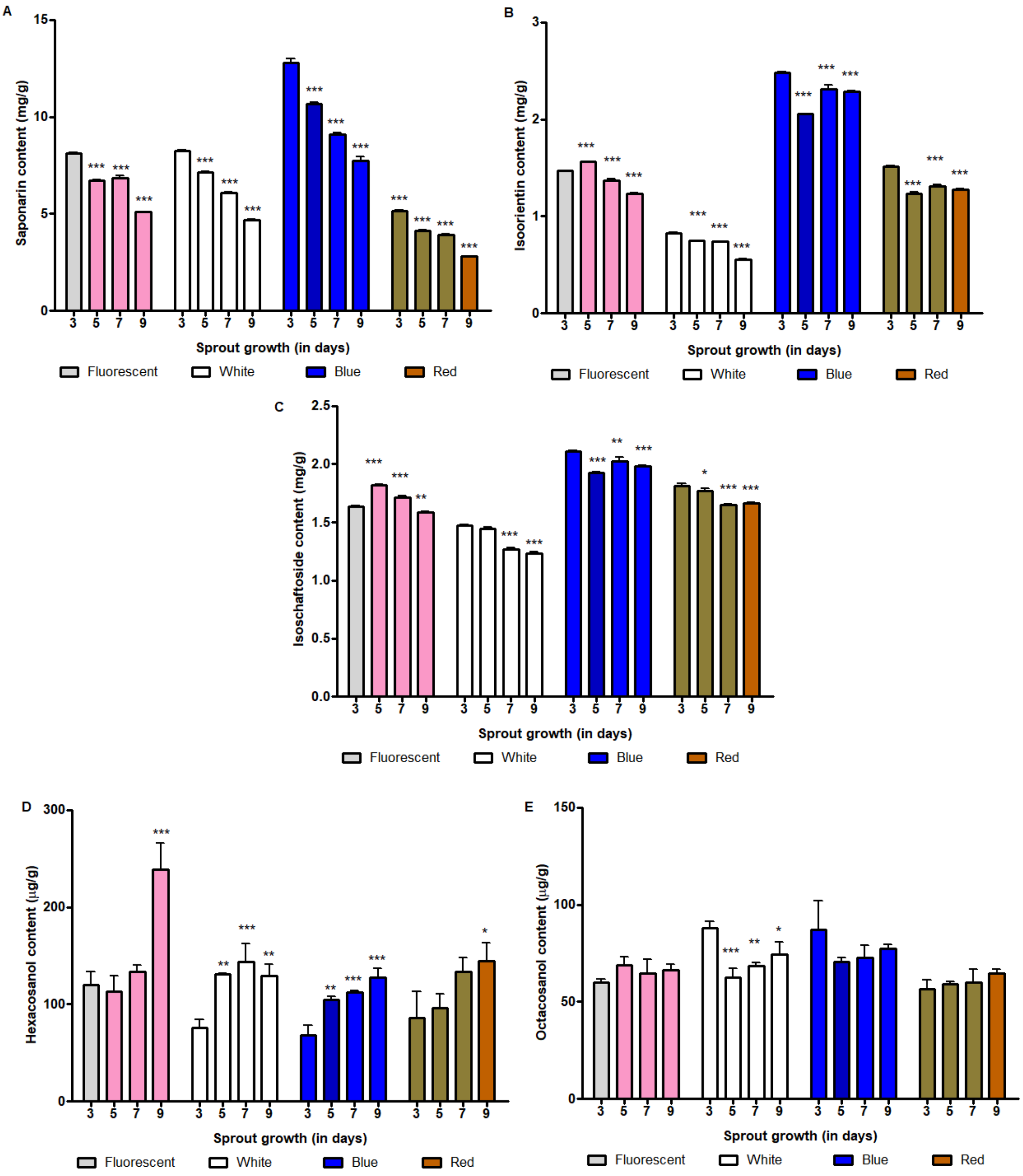

Figure 3. Effect of growth periods on the c-glycosylflavone and policosanol content in fluorescent and LED light irradiated barley and wheat sprouts. A and D represent the Saponarin (mg/g DW) and hexacosanol content (µg/g DW) of barley sprouts. B and C denote the content (mg/g DW) of isoorientin and isoschaftoside in wheat sprouts, while E represents the wheat octacosanol (policosanol) content (µg/g DW). The pink, white, blue, and red colors represent the sprouts treated with fluorescent, white LED, blue LED and red LED light, respectively. * ($P < 0.05$), ** ($P < 0.001$), and *** ($P < 0.0001$) indicate the statistical significance.

Table S1 List of genes, respective gene and protein IDs, primer sequences, and their annealing temperatures used in quantitative RT-PCR assay

Gene	mRNA ID	Protein ID	Primer Sequences	Annealing Temperature ℃

1	HvOGT1	AK375231.1	BAK06426.1	Fp- TGTCGAGATAGTTTGTCGGGTGTG Rp- ATCACGCCGTCGGATGGATATCTG	62
2	HvFNSII	AK375824.1	BAK07019.1	Fp- CGTCGTGCATGTCCCTATGT Rp- TTAATTAGCGTGACAGCGGC	60
3	HvCHS1	AK248641.1		Fp-TACACACGCAGCATCTCACA Rp-CCTTGTCTCACCAGTGACCG	60
4	HvFAR2/ fatty acyl-CoA reductase	HORVU7Hr 1G020270.2		Fp-GGTATACCTCCTACAGCCTGC Rp-CAGATCCGTCTGCCACAAGT	59
5	HvFAR3	AK250407.1		Fp-AAGATGGACGGCAAAGATGGTA RP-GGCCGTACATTAGGACCG	57
6	HvFAR4	AK375080.1		FP-TCTCTGCTTCTCCCTCCCAA Rp-AGCCGGTTGATCCAGTGATG	59
7	HvFAR5	AK375080.1	BAK06275.1	Fp- AAGGGTTGCTTCGACGATGT Rp- CAAAGTTGAATAGCCCGCCG	57
8	HvFAR6	AK374334.1		Fp- ATGGGTCACGCAAAGCAAAC Rp- AGCGTTCACCACCATATCCC	59
9	HvActin	AK362208.1	BAJ93412.1d	Fp-TCGCAACTTAGAAGCACTTCCG Rp- AAGTACAGTGTCTGGATTGGAGGG	--
